# Evaluation of an Artificial Intelligence–Augmented Digital System for Histologic Classification of Colorectal Polyps

**DOI:** 10.1001/jamanetworkopen.2021.35271

**Published:** 2021-11-18

**Authors:** Mustafa Nasir-Moin, Arief A. Suriawinata, Bing Ren, Xiaoying Liu, Douglas J. Robertson, Srishti Bagchi, Naofumi Tomita, Jason W. Wei, Todd A. MacKenzie, Judy R. Rees, Saeed Hassanpour

**Affiliations:** 1 Department of Biomedical Data Science, Geisel School of Medicine, Hanover, New Hampshire; 2 Department of Computer Science, Dartmouth College, Hanover, New Hampshire; 3 Department of Pathology and Laboratory Medicine, Dartmouth-Hitchcock Medical Center, Lebanon, New Hampshire; 4 The Dartmouth Institute for Health Policy and Clinical Practice, Lebanon, New Hampshire; 5 Department of Medicine, Geisel School of Medicine, Hanover, New Hampshire; 6 Section of Gastroenterology, Veterans Affairs Medical Center, White River Junction, Vermont; 7 Department of Community and Family Medicine, Geisel School of Medicine, Hanover, New Hampshire; 8 Department of Epidemiology, Geisel School of Medicine, Hanover, New Hampshire

## Abstract

**Importance:**

Colorectal polyps are common, and their histopathologic classification is used in the planning of follow-up surveillance. Substantial variation has been observed in pathologists’ classification of colorectal polyps, and improved assessment by pathologists may be associated with reduced subsequent underuse and overuse of colonoscopy.

**Objective:**

To compare standard microscopic assessment with an artificial intelligence (AI)–augmented digital system that annotates regions of interest within digitized polyp tissue and predicts polyp type using a deep learning model to assist pathologists in colorectal polyp classification.

**Design, Setting, and Participants:**

In this diagnostic study conducted at a tertiary academic medical center and a community hospital in New Hampshire, 100 slides with colorectal polyp samples were read by 15 pathologists using a microscope and an AI-augmented digital system, with a washout period of at least 12 weeks between use of each modality. The study was conducted from February 10 to July 10, 2020.

**Main Outcomes and Measures:**

Accuracy and time of evaluation were used to compare pathologists’ performance when a microscope was used with their performance when the AI-augmented digital system was used. Outcomes were compared using paired *t* tests and mixed-effects models.

**Results:**

In assessments of 100 slides with colorectal polyp specimens, use of the AI-augmented digital system significantly improved pathologists' classification accuracy compared with microscopic assessment from 73.9% (95% CI, 71.7%-76.2%) to 80.8% (95% CI, 78.8%-82.8%) (*P* < .001). The overall difference in the evaluation time per slide between the digital system (mean, 21.7 seconds; 95% CI, 20.8-22.7 seconds) and microscopic examination (mean, 13.0 seconds; 95% CI, 12.4-13.5 seconds) was –8.8 seconds (95% CI, –9.8 to –7.7 seconds), but this difference decreased as pathologists became more familiar and experienced with the digital system; the difference between the time of evaluation on the last set of 20 slides for all pathologists when using the microscope and the digital system was 4.8 seconds (95% CI, 3.0-6.5 seconds).

**Conclusions and Relevance:**

In this diagnostic study, an AI-augmented digital system significantly improved the accuracy of pathologic interpretation of colorectal polyps compared with microscopic assessment. If applied broadly to clinical practice, this tool may be associated with decreases in subsequent overuse and underuse of colonoscopy and thus with improved patient outcomes and reduced health care costs.

## Key Points

**Question:**

Can an artificial intelligence (AI)–augmented digital system improve classification accuracy of colorectal polyps by pathologists compared with standard microscopic assessment?

**Findings:**

In this diagnostic study including 15 pathologists using microscopic examination and an AI-augmented digital system to interpret 100 slides with colorectal polyp samples, use of the AI-augmented digital system significantly improved pathologists' classification accuracy from 73.9% to 80.8% compared with standard microscopic assessment.

**Meaning:**

These findings suggest that use of an AI-augmented digital system may be associated with improved treatment of patients with colorectal cancer and with improved follow-up surveillance planning to prevent subsequent cancer development.

## Teaser

This diagnostic study evaluates the accuracy and assessment time of an artificial intelligence (AI)–augmented digital system compared with standard microscopic assessment for interpretation of slides with colorectal polyp samples.

## Introduction

Colonoscopy is widely used in the US for colorectal cancer screening and surveillance of colorectal polyps.^1^ More than 15 million colonoscopies are performed in the US each year.^2^ Screening colonoscopy identifies 1 or more adenomas in at least 50% of patients.^3^ When polyps are found, guidelines are used to determine the timing of the next surveillance examination.^4^ However, there is evidence that these recommendations are not followed in clinical practice, leading to substantial overuse and underuse of subsequent colonoscopy.^5,6^ The overuse of colonoscopy is inconvenient for the patient and is associated with increased risk for procedural complications. Overuse also has ramifications for health care costs. Conversely, underuse provides an opportunity for polyps or early cancers to remain undetected and grow, with negative consequences for cancer outcomes. To reduce variation in clinical care recommendations, quality metrics have been established to guide and benchmark endoscopists' performance.^7^

Although the recommendations regarding appropriate surveillance of patients have focused on endoscopists' clinical performance and their application of guidelines,^5,6,8^ histologic interpretation is an equally important factor in determining the appropriate surveillance for an individual patient.^4^ Variability in pathologists’ histopathologic classification of colorectal polyps can result in considerable inconsistencies in the surveillance recommendations given to patients.^9,10,11,12,13,14,15,16^ Moreover, a shortage of pathologists, which is anticipated to continue through 2030, is likely to cause delays and possibly errors in histopathologic characterization of colorectal polyps, a process that is already labor intensive.^2,17^ Thus, an image-analysis system that can quickly and reliably classify different types of colorectal polyps on whole-slide images has the potential to address errors in patient surveillance for colorectal cancer through improved efficiency and accuracy in histopathologic characterization.

The application of deep learning models to the classification of whole-slide pathologic images has been shown to have performance equivalent to that of pathologists in some studies,^18,19,20^ but these studies occurred outside routine clinical care. Assessment of the performance of a deep learning–assisted program can be a challenge. Although prior work has been done to build such systems,^21,22,23^ a comprehensive evaluation of a deep learning system should include many pathologists, conduct a comparison with the standard practice microscope, fully integrate predictions into a whole-slide image viewer, automatically present annotations without requiring pathologists to query for information, and assess efficiencies in real time during routine clinical practice.

Building on an internally and externally validated deep learning model that classifies different types of colorectal polyps,^24^ we developed an artificial intelligence (AI)–augmented digital system for whole-slide images of colorectal polyp tissue samples that classifies and quantifies areas of precancerous tissue. This AI-augmented digital system was compared with the standard practice of microscopic examination used in simulated routine clinical practice to investigate pathologists' accuracy and evaluation time using each method.

## Methods

### Building a Sample Slide Data Set for a Crossover Study

This diagnostic study was conducted from February 10 to July 10, 2020. A convenience sample of 160 slides and whole-slide images was selected. For each of the 4 most frequently identified classes of colorectal polyps (ie, tubular adenoma, tubulovillous or villous adenoma, sessile serrated polyp, and hyperplastic polyp), slides with hematoxylin and eosin–stained formalin-fixed, paraffin-embedded colorectal polyp samples were collected according to the classification that appeared in the Dartmouth-Hitchcock Medical Center (DHMC) electronic health records as determined by the local pathologists.^24^ The selection was made in reverse chronological order as determined by the date entered in the electronic health record starting on December 31, 2019, and was continued until 40 candidate slides had been acquired for each of 4 pathologic classifications. Slides were only eligible for inclusion if every specimen on a single slide belonged to the same polyp and no 2 specimens had been obtained from the same patient. This study was approved by the Dartmouth-Hitchcock Health institutional review board with a waiver of the requirement of informed consent from the patients because deidentified slides were used. All pathologist readers who participated in the study provided written informed consent. This study followed the Transparent Reporting of a Multivariable Prediction Model for Individual Prognosis or Diagnosis (TRIPOD) reporting guideline.

To establish the gold standard classifications based on the 2019 World Health Organization classification of digestive tumors,^25,26^ all 160 retrieved candidate slides were independently reviewed by 3 gastrointestinal tract (GI) pathologists who were blinded to the original classification of the slides and their associated clinical notes. Disagreements (26 slides) were resolved by consensus achieved through discussion among the 3 GI pathologists. The final class distribution for the 160 slides was 41 hyperplastic polyps, 35 sessile serrated polyps, 39 tubulovillous or villous adenomas, and 45 tubular adenomas. Based on the gold standard classification, 25 slides from each class were randomly selected for inclusion during the study for a total of 100 slides. Another 10 slides (3 tubular adenomas, 2 tubulovillous or villous adenomas, 2 hyperplastic polyps, and 3 sessile serrated polyps) were randomly selected from the remaining 60 slides to serve as example slides for training pathologists in use of the digital system.

### Whole-Slide Image Inference Using Deep Learning

All the slides were scanned at a magnification of 40× using a Leica Aperio AT2 scanner (Leica Biosystems). The resulting whole-slide images were fed to a ResNet-18 neural network,^27^ which was developed and validated to classify colorectal polyps into 4 classes (ie, tubular adenoma, tubulovillous or villous adenoma, sessile serrated polyp, and hyperplastic polyp) with an independent set of 508 slides from DHMC and was previously validated with 238 external slides from 24 different institutions.^24,28^ The model used a sliding-window approach in which predictions were made on patches of 224 × 224 pixels. These predictions were then used to calculate the percentage of patches, a proxy for the percentage of area, attributed to each class in the whole-slide image. The percentage of patches for each class was then used in a decision tree to determine the overall class of the whole-slide image.^24^ For our digital system, we extracted the percentage of patches attributed to each class, the coordinates for the regions of interest highlighted by the classifier for each class, and the whole-slide image prediction.

### AI–Augmented Digital System

**Figure 1.**
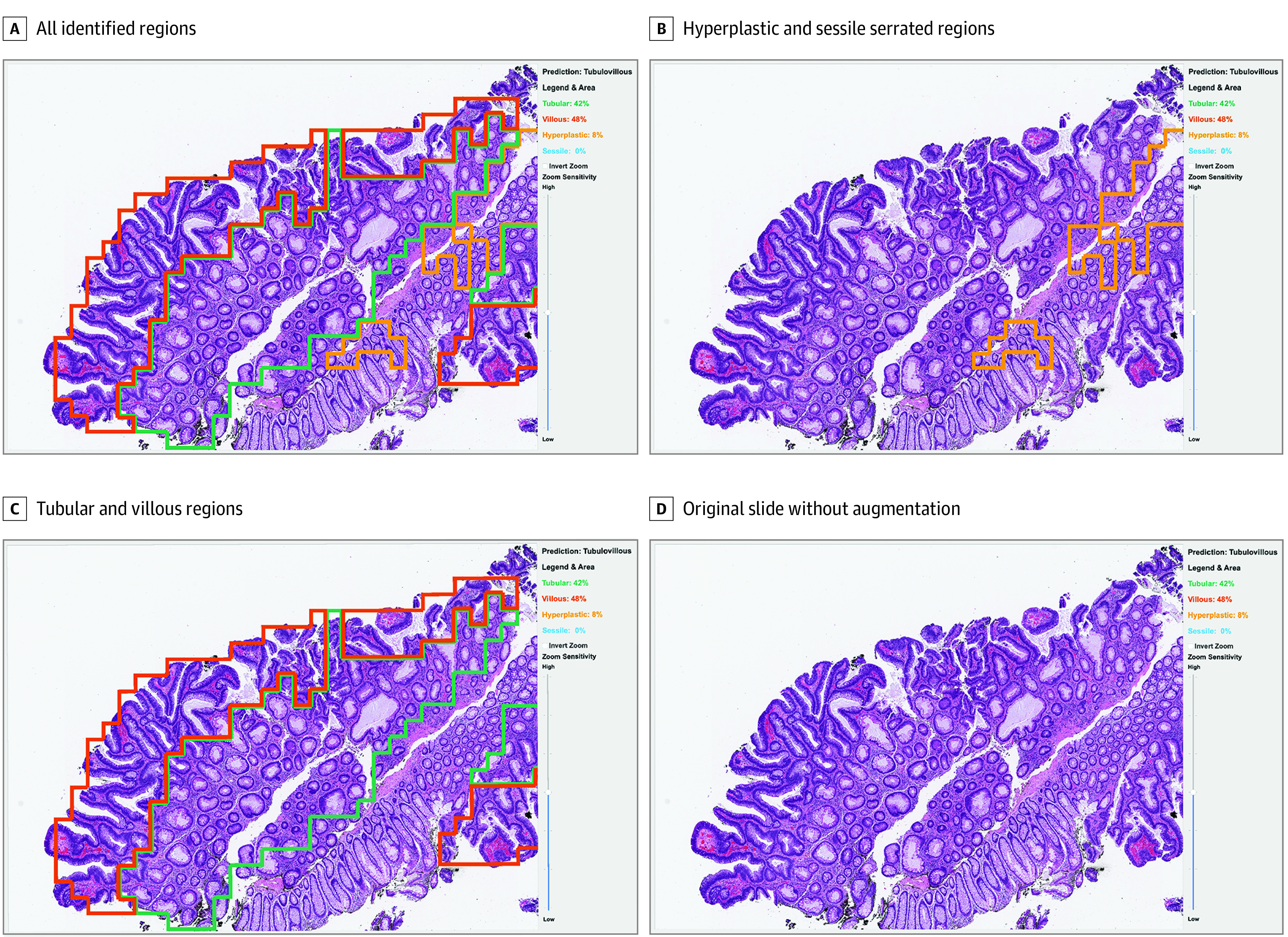
User Interface of the Artificial Intelligence–Augmented Digital System The graphical user interface displayed the whole-slide image, the deep learning model's prediction, and the percentage of area attributed to the tubular, villous, hyperplastic, and sessile serrated components. Users had the option to cycle through preprogrammed combinations of regions of interest.

We developed a user interface that displayed regions of interest highlighted by the deep learning classifier to examine the effects of using an AI-augmented diagnostic assistance tool in clinical practice. The regions of each histologic type were color coded as explained in a legend contained in a sidebar on the right side of the display. This sidebar also included the predicted classes of the whole-slide images as identified by the classifier and the percentage of patches attributed to each class to aid pathologists through quantification instead of having them rely on visual estimations. The area quantification and predicted class were displayed throughout the review, and pathologists had the option of viewing each whole-slide image without the regions of interest. This user interface is shown in Figure 1. The digital system was displayed on a 34-inch Dell U3417W monitor (Dell Technologies) with a resolution of 3440 × 1440.

### Randomized Crossover Study Design

The study was conducted at DHMC, an academic medical center, and Cheshire Medical Center, a DHMC-affiliated community hospital. The 2 primary outcomes of interest were accuracy and time of evaluation when a pathologist used a standard practice microscope compared with when a pathologist used the AI-augmented digital system. A total of 15 pathologists were included in the study: 8 board-certified pathologists (6 from DHMC and 2 from Cheshire Medical Center) and 7 DHMC pathology residents (1 in postgraduate year [PGY] 4, 1 in PGY 3, 3 in PGY 2, and 2 in PGY 1) (eTable 1 in the Supplement). Of the 6 participating DHMC pathologists, 2 were GI pathologists, but they were not the same GI pathologists who established the aforementioned gold standard classifications.

**Figure 2.**
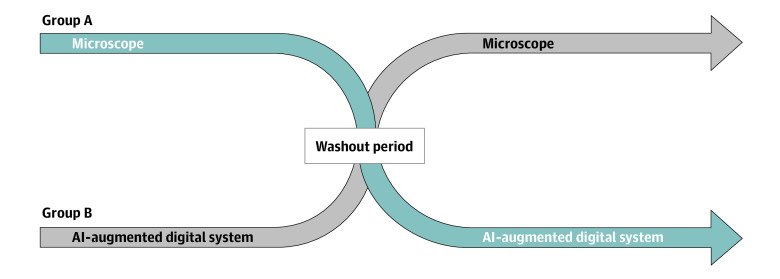
Randomized Crossover Design of Study Each pathologist was randomly assigned to review 100 slides with a given device. After a washout period of at least 12 weeks, each pathologist reviewed the same set of slides in a different (randomly shuffled) order using the other device.

The study had a randomized crossover design (Figure 2). Each pathologist participated in 2 sessions in which a shuffled set of 100 slides was reviewed by using either the traditional microscope or the digital system. Eight pathologists were randomly assigned to first use the microscope, and the remaining 7 were assigned to first use the digital system. After a washout period of at least 12 weeks to minimize recall bias, each pathologist reviewed the same slides but in a shuffled order and using the other tool. Pathologists were instructed to specifically classify each slide as a tubular adenoma, a tubulovillous or villous adenoma, a sessile serrated polyp, or a hyperplastic polyp. They were encouraged to take as much time as necessary to be confident in their classification as they would do in a clinical setting.

Study pathologists were blinded to any information regarding the number or distribution of polyp classes on the slides, clinical histories, follow-up information, and any previous polyp classifications associated with each patient from whom a specimen was obtained. Before starting the digital system session, pathologists watched a 5-minute training video, read a brief summary of how the predictions were generated, and practiced using a set of 10 slides to become familiar with the system. Time of evaluation was defined as the time between the pathologist starting and stopping viewing a slide and was measured using a timer that was embedded in the digital system during the digital session and by a video camera during the microscope session. The video was subsequently reviewed by research staff to identify the start and stop time of each slide.

Before the study was conducted, pathologists completed a survey describing their clinical experience level (see eTable 2 in the Supplement). After the digital session, pathologists completed a survey to provide feedback on the digital system using the System Usability Scale^29^ (eTable 3 in the Supplement) and the Paas mental-effort scale^30^ (eTable 4 in the Supplement) and by providing written comments (eTable 5 in the Supplement).

### Statistical Analysis

The overall accuracy, defined as the proportion of correctly classified polyps, was calculated for all 15 pathologists (2 GI pathologists, 6 non-GI pathologists, and 7 pathology residents) for use of the microscope and the digital system. The mean per-class accuracy and the overall accuracy by pathologist training level were computed for use of the microscope and the digital system.

Statistical differences in the accuracy of pathologists' classifications during use of a microscope or the digital system were assessed with a paired *t* test. A logistic mixed-effects model was developed to assess accuracy with the fixed effects of the digital system, the pathologist’s training level, and an interaction between reading order (ie, whether the pathologist was assigned to the microscope or AI-augmented digital system first) and period (ie, whether the reading was made during the first or second round of the study); the overall accuracy for each slide and the overall accuracy for each pathologist were considered as random effects. Fleiss κ was calculated to measure interrater reliability of classifications made using the microscope or the digital system to assess diagnostic consistency between pathologists using each method.

To assess the time of evaluation and the mean per-class time, the mean evaluation time by pathologist training level was calculated when pathologists used the microscope and the digital system. A paired *t* test was also used to evaluate whether at the individual level there was a significant difference between a given pathologist's mean time of evaluation with the microscope vs with the digital system. We assessed time of evaluation using a linear mixed-effects model in which the digital system was considered as a fixed effect and the mean time required to read each slide and the mean time for each pathologist were considered random effects. The mean times of evaluation for use of the microscope and the digital system were calculated for each quintile of slides and monitored for changes. Statistical analyses were completed in R statistical software, version 4.0.0 (R Foundation for Statistical Computing). All analyses used an α of .05, and measures are presented with 95% CIs.

## Results

### Accuracy and Interrater Reliability

Overall, the digital system user interface increased the accuracy of reading of pathologic findings in a sample of 1500 readings conducted by the 15 pathologists. Specifically, among the 15 pathologists, accuracy was better with the digital system (80.8%; 95% CI, 78.8%-82.8%) compared with conventional assessment with the microscope (73.9%; 95% CI, 71.7%-76.2%). Accuracy was most improved for identification of a tubulovillous or villous adenoma, for which the digital system improved reading by 21.3% (95% CI, 15.3%-27.3%).

The logistic mixed-effects model for accuracy showed that the digital system increased the odds of a correct classification by a factor of 1.80 (95% CI, 1.45-2.22; *P* < .001), and the aforementioned training levels and order did not have a significant association with accuracy. The deep learning model without a pathologist user achieved an accuracy of 87.0% (95% CI, 82.2%-91.7%) overall. The accuracy of each pathologist with each device is shown in eFigure 1 and eTable 6 in the Supplement. Table 1 shows the mean per-class accuracy and the overall accuracy by pathologist training level for use of the microscope and use of the digital system. The accuracy was greatest and the difference in accuracy lowest for GI pathologists (microscope: 82.5% [95% CI, 77.2% to 87.8%]; digital: 86.5% [95% CI, 81.8% to 91.2%]; difference: 4.0% [95% CI, –2.6% to 10.6%]), and the accuracy was lowest and the difference in accuracy greatest for residents (microscope: 70.0% [95% CI, 66.6%-73.4%]; digital: 78.9% [95% CI, 75.8%-81.9%]; difference: 8.9% [95% CI, 5.0%-12.7%]).

**Table 1.** Pathologists' Accuracy by Review Modality

Variable	Accuracy, % (95% CI)		Difference, % (95% CI)	*P* value
	Microscope	Digital		
Polyp type^a^				
Tubulovillous or villous adenoma	49.6 (44.5 to 54.7)	70.9 (66.3 to 75.5)	21.3 (15.3 to 27.3)	<.001
Sessile serrated polyp	63.5 (58.6 to 68.3)	66.1 (61.3 to 70.9)	2.6 (–2.8 to 8.1)	.34
Tubular adenoma	89.6 (86.5 to 92.7)	91.2 (88.3 to 94.1)	1.6 (–2.3 to 5.5)	.42
Hyperplastic polyp	93.1 (90.5 to 95.6)	94.9 (92.7 to 97.2)	1.9 (–1.6 to 5.3)	.29
All	73.9 (71.7 to 76.2)	80.8 (78.8 to 82.8)	6.9 (4.4 to 9.4)	<.001
Training level^b^				
GI pathologist (n = 2)	82.5 (77.2 to 87.8)	86.5 (81.8 to 91.2)	4.0 (–2.6 to 10.6)	.24
Non-GI pathologist (n = 6)	75.7 (72.2 to 79.1)	81.1 (78.0 to 84.3)	5.5 (1.7 to 9.2)	.004
Resident (n = 7)	70.0 (66.6 to 73.4)	78.9 (75.8 to 81.9)	8.9 (5.0 to 12.7)	<.001
All (n = 15)	73.9 (71.7 to 76.2)	80.8 (78.8 to 82.8)	6.9 (4.4 to 9.4)	<.001

Abbreviation: GI, gastrointestinal.

^a^ For each class, 375 readings were conducted using each method (microscope or digital).

^b^ One hundred readings were conducted using each method (microscope or digital) per pathologist.

The Fleiss κ, a measure of interrater agreement, for the classifications by the 15 pathologists was 0.61 when they used the microscope and 0.69 when they used the digital system. In comparison, the Fleiss κ for the 3 GI pathologists' classifications, encompassing the gold standard classifications, was 0.92.

### Time

The mean time of evaluation for all pathologists was longer when the digital system was used (mean, 21.7 seconds; 95% CI, 20.8-22.7 seconds) than when the microscope was used (mean, 13.0 seconds; 95% CI, 12.4-13.5 seconds) (difference: –8.8 seconds; 95% CI, –9.8 to –7.7 seconds) (Table 2 and eFigure 2 in the Supplement). This difference decreased in association with greater pathologist training level (GI pathologist: –11.9 seconds [95% CI, –13.9 to –9.9 seconds]; resident: –9.3 seconds [95% CI, –11.0 to –7.6 seconds]) (Table 2). The slower evaluation speed was confirmed within individuals (paired *t* test: mean, –8.8 seconds; 95% CI, –11.8 to –6.0; *P* < .001) and by the linear mixed-effect model (–8.8 seconds; 95% CI, –9.8 to –7.7 seconds). The mean time of evaluation for all pathologists when the microscope was used decreased during the second, fourth, and fifth quintiles but increased during the third quintile. Overall, the mean time of evaluation decreased by 2.8 seconds (95% CI, 0.8-4.6 seconds) from the first 20 slides (14.5 seconds; 95% CI, 12.8-16.0 seconds) to the last 20 slides (11.7 seconds; 95% CI, 11.7-12.9 seconds) (eFigure 3 in the Supplement). The mean time of evaluation for all pathologists when the digital system was used decreased consistently after each quintile of slides and by 12.4 seconds (95% CI, 9.3-15.7 seconds) from the first 20 slides (28.9 seconds; 95% CI, 25.9-32.0 seconds) to the last 20 slides (16.5 seconds; 95% CI, 15.0-18.0 seconds). The difference between the time of evaluation on the last set of 20 slides for all pathologists when using the microscope and the digital system was 4.8 seconds (95% CI, 3.0-6.5 seconds) (eFigure 3 in the Supplement).

**Table 2.** Pathologists' Time of Evaluation by Review Modality

Variable	Time of evaluation, mean (95% CI), s		Difference (95% CI), s	*P* value
	Microscope	Digital		
Polyp type^a^				
Tubulovillous or villous adenoma	9.2 (8.5 to 9.9)	15.3 (13.5 to 17.1)	–6.1 (–8.0 to –4.2)	<.001
Sessile serrated polyp	15.7 (14.6 to 16.9)	28.5 (26.4 to 30.5)	–12.7 (–15.0 to –10.4)	<.001
Tubular adenoma	10.8 (9.8 to 11.8)	20.0 (18.2 to 21.9)	–9.2 (–11.1 to –7.3)	<.001
Hyperplastic polyp	16.1 (14.7 to 17.5)	23.1 (21.3 to 24.8)	–7.0 (–9.0 to –4.9)	<.001
All	13.0 (12.4 to 13.5)	21.7 (20.8 to 22.7)	–8.8 (–9.8 to –7.7)	<.001
Training level^b^				
GI pathologist (n = 2)	7.6 (6.7 to 8.4)	19.5 (17.3 to 21.7)	–11.9 (–13.9 to –9.9)	<.001
Non-GI pathologist (n = 6)	12.2 (11.3 to 13.0)	19.2 (17.8 to 20.6)	–7.0 (–8.5 to –5.5)	<.001
Resident (n = 7)	15.2 (14.3 to 16.1)	24.5 (23.0 to 26.0)	–9.3 (–11.0 to –7.6)	<.001
All (n = 15)	13.0 (12.4 to 13.5)	21.7 (20.8 to 22.7)	–8.8 (–9.8 to –7.7)	<.001

Abbreviation: GI, gastrointestinal.

^a^ For each class, 375 readings were conducted using each method (microscope or digital).

^b^ One hundred readings were conducted using each method (microscope or digital) per pathologist.

### Usability

The results of the System Usability Scale for the digital system are summarized in eFigure 4 in the Supplement. The mean score for the System Usability Scale for the digital system was 68.2 (95% CI, 61.3-75.0), which translates to a good usability.^31^ The individual scores are shown in eFigure 5 in the Supplement. Moreover, 7 of the 15 pathologists stated that they would use a version of this tool to evaluate slides routinely, and another 4 pathologists stated that they would possibly use a version of this tool to evaluate slides routinely. The mean (SD) Paas mental-effort rating, which ranges from “very, very low mental effort” (1) to “very, very, high mental effort” (9), was 5 (1.3), corresponding to “neither low nor high mental effort.”^30^

### Error Analysis

**Figure 3.**
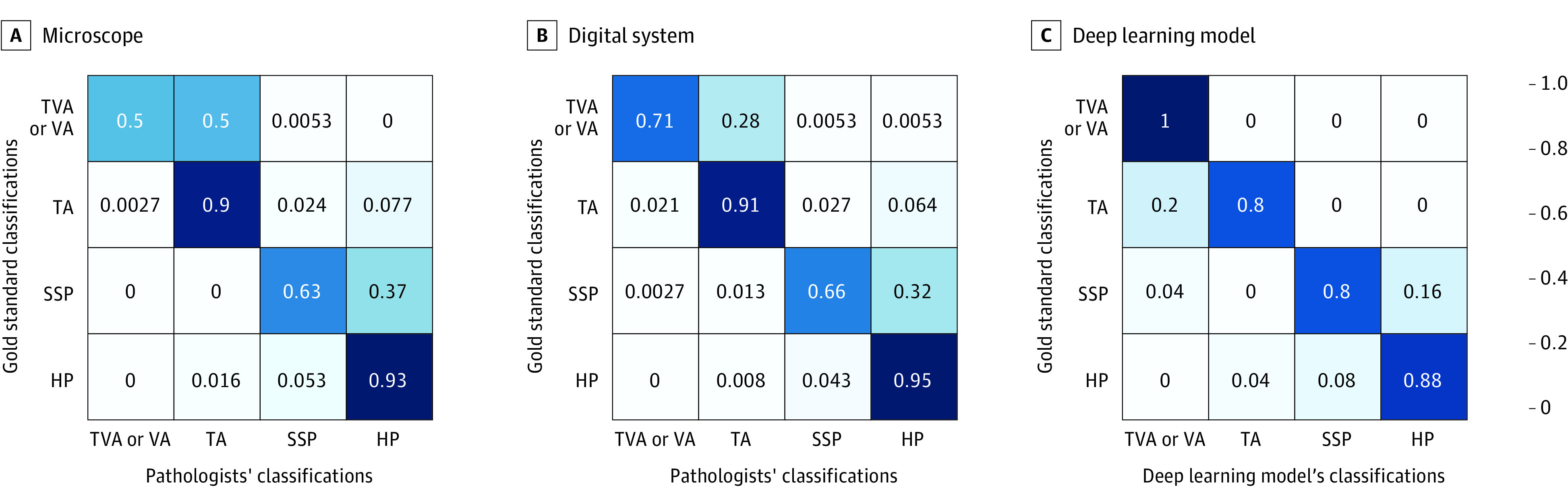
Confusion Matrixes for Pathologists' Errors By Review Modality For each class, 375 readings were conducted by pathologists using each method (microscope or digital system). The deep learning model also independently classified the same set of slides. HP indicates hyperplastic polyp; SSP, sessile serrated polyp; TA, tubular adenoma; TVA, tubulovillous adenoma; VA, villous adenoma.

The mean accuracy increased by 8.9% (95% CI, 6.3%-11.5%) with use of the digital system compared with use of the microscope on the subset of 87 slides in which the deep learning model's prediction was correct. Pathologist accuracy decreased by 6.7% (95% CI, −0.7% to 14.2%) with the digital system compared with the microscope on the subset of 13 slides in which the deep learning model's prediction was incorrect. Confusion matrixes were constructed using all classifications made with the microscope and digital system. Comparison of the confusion matrixes revealed that the use of the digital system reduced the overall number of misclassifications, but the pattern of mistakes made by the pathologists overall was similar. For example, in our study, the digital system reduced the misclassification of tubulovillous or villous adenomas as tubular adenomas from 49.9% (95% CI, 44.7%-55.1%) to 28% (95% CI, 23.5%-32.8%), the misclassification of sessile serrated polyps as hyperplastic polyps from 36.5% (95% CI, 31.6%-41.6%) to 32.2% (95% CI, 27.5%-37.2%), and the misclassification of hyperplastic polyps as sessile serrated polyps from 5.3% (95% CI, 3.3%-8.1%) to 4.3% (95% CI, 2.5%-6.9%) (Figure 3).

A review of discrepant classifications revealed that misclassifying tubular adenomas as tubulovillous adenomas was often attributable to the deep learning model classifying aligned elongated crypts as villous architecture. In addition, the model did not detect branching crypts in specimens in which sessile serrated polyps were incorrectly classified as hyperplastic polyps. Other erroneous classifications were borderline; the model correctly annotated tubular, villous, hyperplastic, and serrated patterns, and the polyps could have been called either tubular adenomas or tubulovillous adenomas or hyperplastic or sessile serrated polyps. There was a bloody section that the model deemed to be a villous region, leading to a misclassification of a sessile serrated polyp as a tubulovillous adenoma.

## Discussion

This diagnostic study evaluated the effect of AI assistance on pathologist performance in clinical practice, comparing accuracy, speed, and consistency using a crossover model with an internally and externally validated deep learning model. The augmented digital system substantially improved pathologist accuracy (80.8%) compared with standard microscopic assessment (73.9%); the deep learning model alone was more accurate (87.0%) than the pathologists using the microscope or the augmented digital system. For patients with polyps, subsequent colorectal cancer risk and follow-up recommendations depend on the histopathologic characterization of the identified polyps. However, accurate characterization is challenging and varies markedly even among expert GI pathologists.^9,10,11,12,13,14,15,16,32^ We demonstrated that an AI-augmented digital system improved the accuracy of pathologic classification of polyps. Use of this tool might lead to an increased frequency of patients receiving appropriate follow-up surveillance recommendations to prevent subsequent cancer development and reduce colonoscopy overuse.

Improvements in reading of pathologic findings by an AI-augmented digital system may affect surveillance recommendations in 2 ways—each with advantages to patients and the health care system. First, improved readings of pathologic findings may reduce the underrating of lesions suggestive of more advanced disease on histologic examination. In these cases, accurate reading of pathologic findings may be associated with shortened surveillance intervals and a reduced likelihood that cancer will progress unnoticed. Accurate reading of pathologic findings may also be associated with increased surveillance intervals for patients less likely to benefit from intense surveillance. Based on the US Multi-Society Task Force on Colorectal Cancer guidelines,^4^ the frequency of surveillance colonoscopy can be increased by up to 7 years when hyperplastic polyps are misclassified as sessile serrated polyps. Thus, in these cases, accurate reading of pathologic findings may be associated with a reduced burden on the health care system and on individual patients who are less likely to benefit from more frequent surveillance examinations.

However, in general, pathologists using the microscope or the digital system did not outperform the stand-alone deep learning model, reflecting the ability of deep learning models to match or outperform pathologists.^18,20,22,24,33,34^ Although this result might suggest that AI alone should be incorporated into reading of pathologic findings in clinical practice, there are technical and regulatory hurdles to such implementation. Furthermore, pathologists' knowledge and experience bring value, especially in cases in which the deep learning model is incorrect. Thus, refinement of both the underlying deep learning model and the presentation of its information may lead to further gains when an AI-augmented system is used.

With respect to time, pathologists completed readings more quickly when they used a microscope than when they used the digital system. We suspect that the time difference could be lowered with further training and practice with the digital system given a downward trend in the mean time of evaluation as pathologists read more slides and that the difference between the digital system and microscope for the last quintile of slides was only 4.8 seconds. Still, the small addition of time must be balanced against increases in accuracy with use of the digital system. In addition, overall, the mean System Usability Scale score for the digital system indicated that the usability was good, which is encouraging considering the short training and use period for the system.

### Limitations

This study has limitations. First, the digital system did not use clinical information, including the location of the biopsy, which can be an important factor when a classification is being made, particularly for serrated lesions. Combining clinical metadata and medical images can involve training multiple deep learning models and is further complicated by automatic information extraction from electronic health records.^35,36^ In this study, we used 1 slide per patient, but in practice, multiple slides are often available for each patient to make the final classification. Our future work will include relevant clinical notes within the digital system and multiple slides per patient. In addition, we plan to extend our model to other clinically important histologic classes such as traditional serrated adenomas and high-grade dysplasia.

## Conclusions

In this diagnostic study, an AI-augmented digital system improved the accuracy of histopathologic interpretation of colorectal polyps compared with microscopic assessment. Wide adoption of this tool in clinical practice may be associated with limited overuse and underuse of subsequent surveillance colonoscopy; reduced stress, financial burden, and complications from unnecessary procedures; and prevention of delayed diagnosis of cancer.

## Notes

Supplement. **eFigure 1.** Per-pathologist Accuracy on a Set of 100 Slides **eFigure 2.** Violin Plots and Box Plots Displaying the Time of Evaluation for 100 Slides for Each Pathologist **eFigure 3.** Changes in the Average Time of Evaluation When Using a Microscope and the Digital System for Each Quintile of Slides **eFigure 4.** Likert Scale Results From the System Usability Scale Survey for the Digital System **eFigure 5.** System Usability Scale Score Indicating the Overall Usability of the Digital System for Each Pathologist **eTable 1.** Crossover Study Participants **eTable 2.** The Pre-study Survey, Which Was Completed by Each Pathologist **eTable 3.** The System Usability Scale Is a Likert Scale With the Following Rankings: Strongly Disagree, Disagree, Neutral, Agree, and Strongly Agree **eTable 4.** The PAAS Mental-Effort Scale Is a Likert Scale With the Following Rankings: Very, Very Low Mental Effort; Very Low Mental Effort; Rather Low Mental Effort; Low Mental Effort; Neither Low Nor High Mental Effort; Rather High Mental Effort; High Mental Effort; Very High Mental Effort; and Very, Very High Mental Effort **eTable 5.** Questions That Promoted Free-Prose Feedback **eTable 6.** Per Class Accuracy, Sensitivity, and Specificity Across Both Devices for Each Pathologist
