# Supplementary material for: Evaluation of an Artificial Intelligence–Augmented Digital System for Histologic Classification of Colorectal Polyps
Source: JAMA Netw Open. 2021 Nov 18;4(11):e2135271. doi: 10.1001/jamanetworkopen.2021.35271 (PMC8603082; doi:10.1001/jamanetworkopen.2021.35271)
Supplement: Supplement. — eFigure 1. Per-pathologist Accuracy on a Set of 100 Slides eFigure 2. Violin Plots and Box Plots Displaying the Time of Evaluation for 100 Slides for Each Pathologist eFigure 3. Changes in the Average Time of Evaluation When Using a Microscope and the Digital System for Each Quintile of Slides eFigure 4. Likert Scale Results From the System Usability Scale Survey for the Digital System eFigure 5. System Usability Scale Score Indicating the Overall Usability of the Digital System for Each Pathologist eTable 1. Crossover Study Participants eTable 2. The Pre-study Survey, Which Was Completed by Each Pathologist eTable 3. The System Usability Scale Is a Likert Scale With the Following Rankings: Strongly Disagree, Disagree, Neutral, Agree, and Strongly Agree eTable 4. The PAAS Mental-Effort Scale Is a Likert Scale With the Following Rankings: Very, Very Low Mental Effort; Very Low Mental Effort; Rather Low Mental Effort; Low Mental Effort; Neither Low Nor High Mental Effort; Rather High Mental Effort; High Mental Effort; Very High Mental Effort; and Very, Very High Mental Effort eTable 5. Questions That Promoted Free-Prose Feedback eTable 6. Per Class Accuracy, Sensitivity, and Specificity Across Both Devices for Each Pathologist [file jamanetwopen-e2135271-s001.pdf]

Supplementary Online Content

Nasir-Moin M, Suriawinata AA, Ren B, et al. Evaluation of an artificial intelligence–augmented digital system for histologic classification of colorectal polyps. *JAMA Netw Open*. 2021;4(11):e2135271. doi:10.1001/jamanetworkopen.2021.35271

eFigure 1. Per-pathologist Accuracy on a Set of 100 Slides
eFigure 2. Violin Plots and Box Plots Displaying the Time of Evaluation for 100 Slides for Each Pathologist
eFigure 3. Changes in the Average Time of Evaluation When Using a Microscope and the Digital System for Each Quintile of Slides
eFigure 4. Likert Scale Results From the System Usability Scale Survey for the Digital System
eFigure 5. System Usability Scale Score Indicating the Overall Usability of the Digital System for Each Pathologist
eTable 1. Crossover Study Participants
eTable 2. The Pre-study Survey, Which Was Completed by Each Pathologist
eTable 3. The System Usability Scale Is a Likert Scale With the Following Rankings: Strongly Disagree, Disagree, Neutral, Agree, and Strongly Agree
eTable 4. The PAAS Mental-Effort Scale Is a Likert Scale With the Following Rankings: Very, Very Low Mental Effort; Very Low Mental Effort; Rather Low Mental Effort; Low Mental Effort; Neither Low Nor High Mental Effort; Rather High Mental Effort; High Mental Effort; Very High Mental Effort; and Very, Very High Mental Effort
eTable 5. Questions That Promoted Free-Prose Feedback
eTable 6. Per Class Accuracy, Sensitivity, and Specificity Across Both Devices for Each Pathologist

This supplementary material has been provided by the authors to give readers additional information about their work.

© 2021 Nasir-Moin M et al. *JAMA Network Open*.

Supplemental Figures

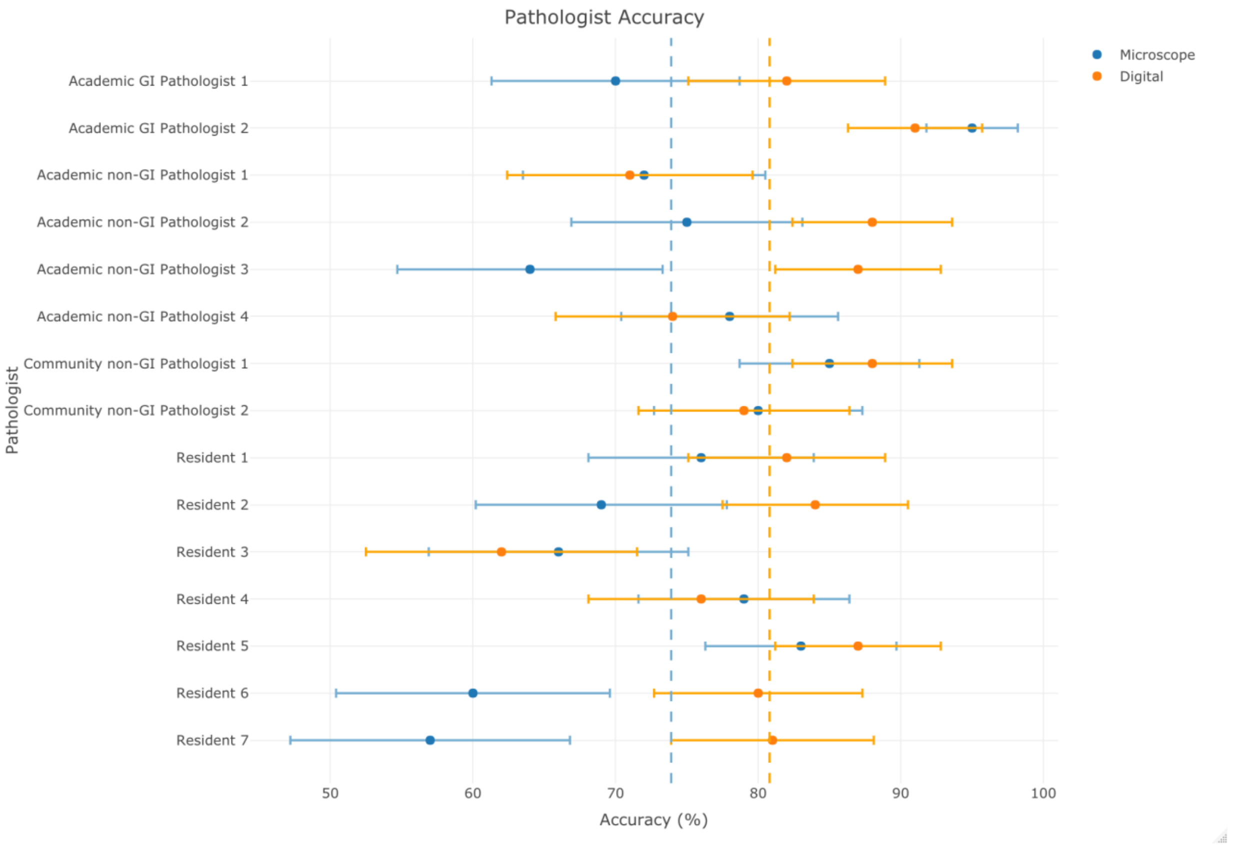

eFigure 1. Per-pathologist accuracy on a set of 100 slides. The overall accuracy and 95% confidence interval for each pathologist is shown using the microscope (blue) and digital system (orange). The orange and blue dashed vertical lines indicate the overall accuracy for all pathologists for the digital system, and the microscope, respectively.

© 2021 Nasir-Moin M et al. *JAMA Network Open*.

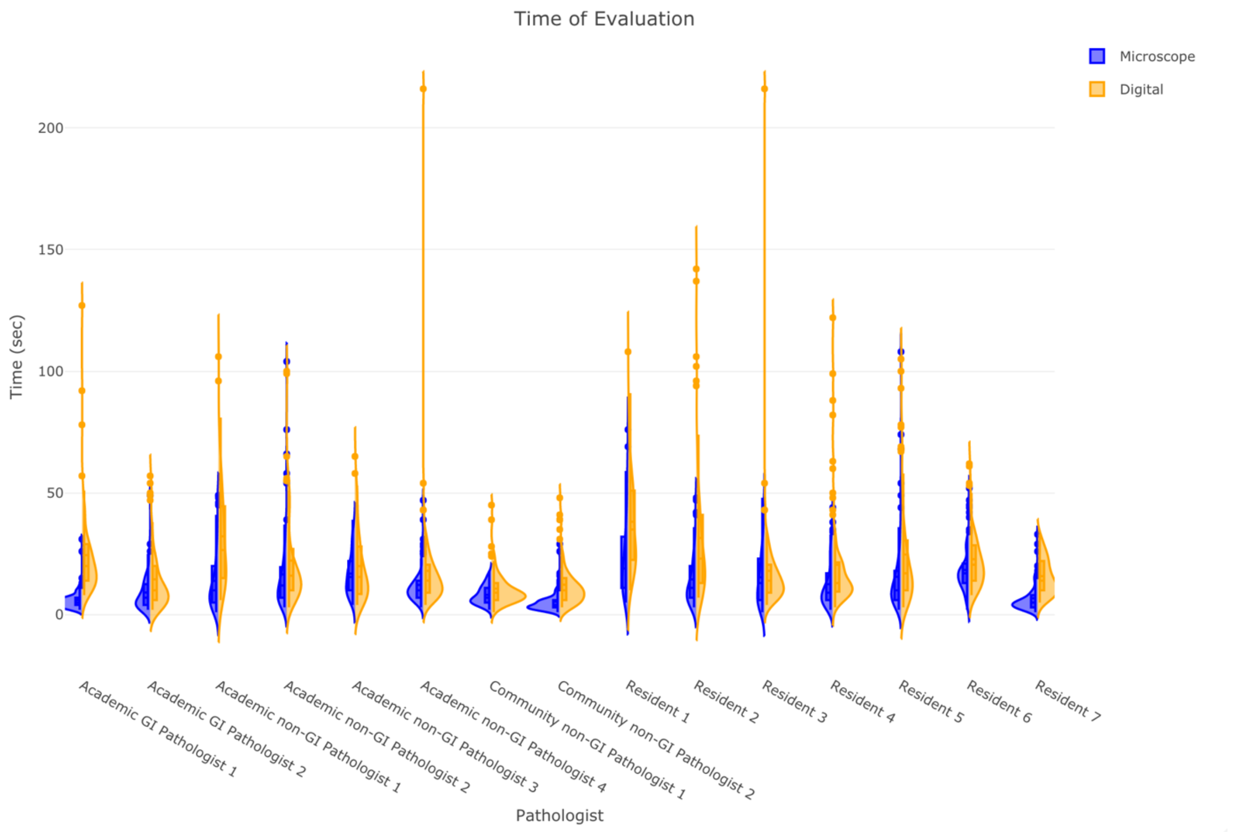

eFigure 2. Violin plots and box plots displaying the time of evaluation for 100 slides for each pathologist. The orange and blue dashed vertical lines indicate the average time of the assessment for the digital system and the microscope, respectively.

© 2021 Nasir-Moin M et al. *JAMA Network Open*.

a)

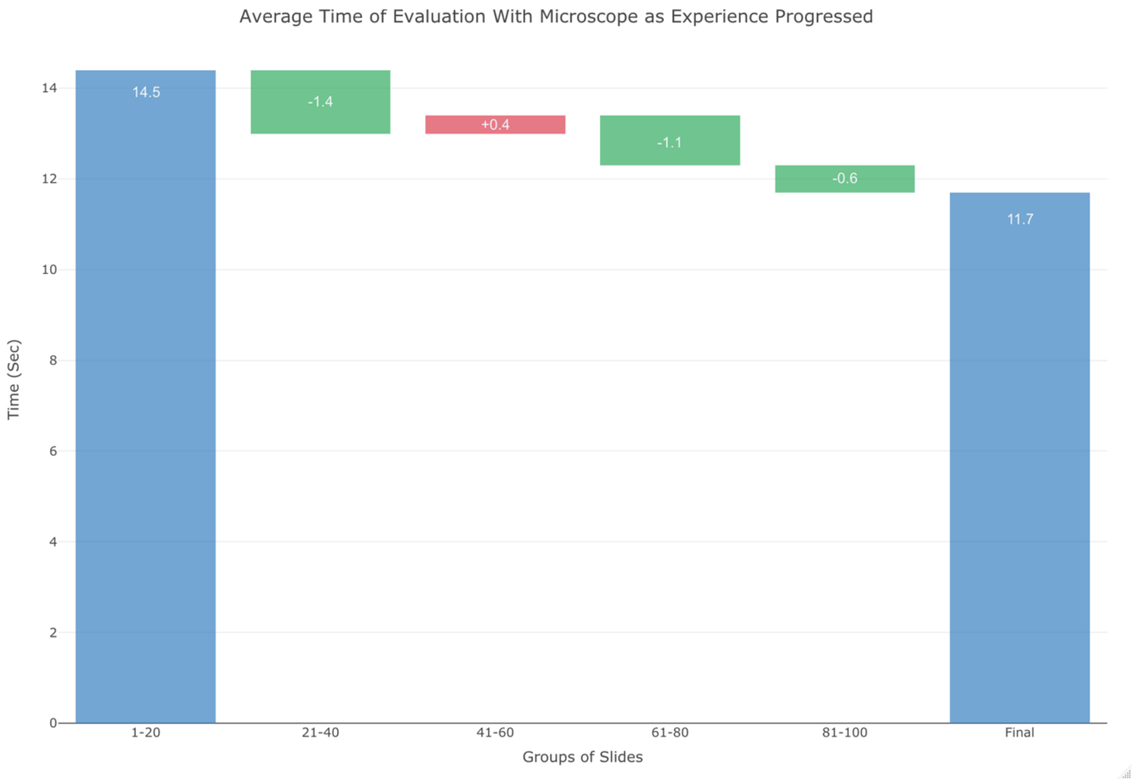

b)

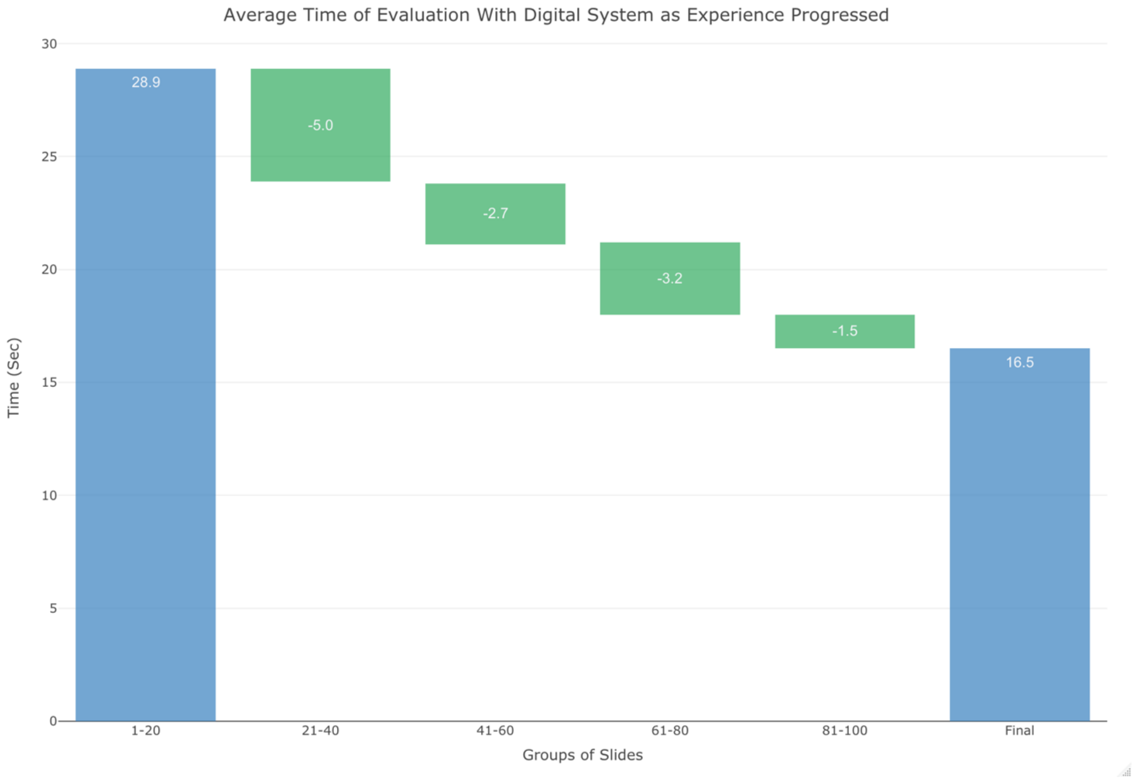

© 2021 Nasir-Moin M et al. *JAMA Network Open*.

eFigure 3. Changes in the average time of evaluation when using a microscope and the digital system for each quintile of slides.

© 2021 Nasir-Moin M et al. *JAMA Network Open*.

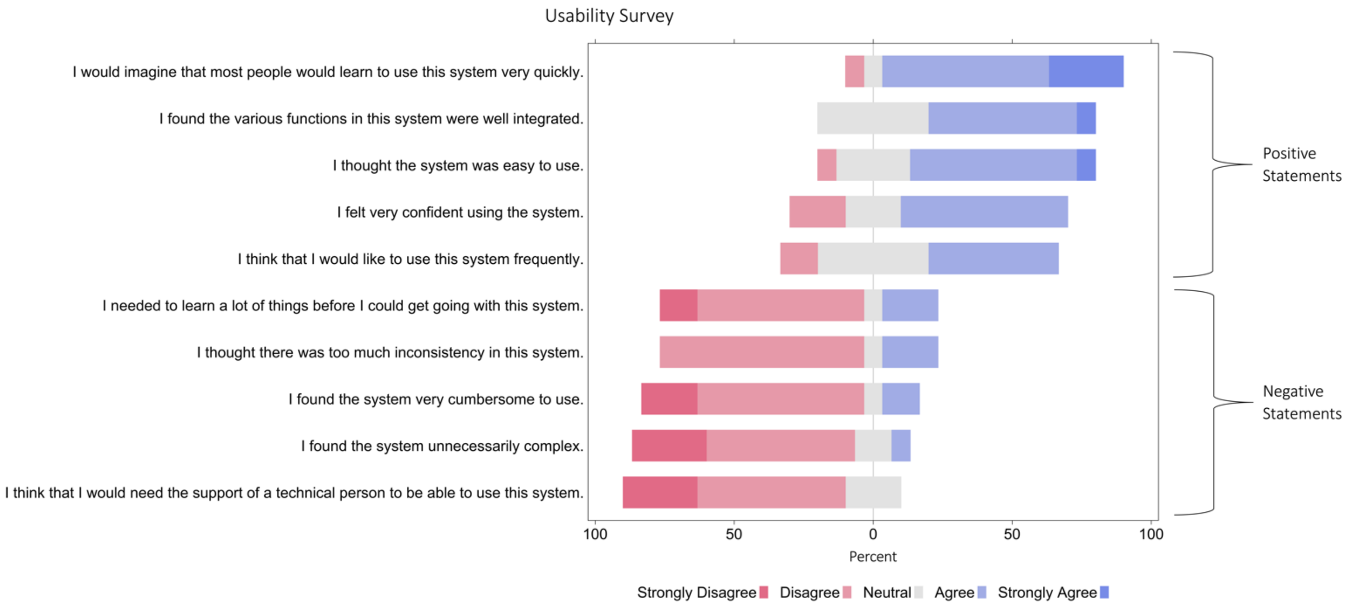

eFigure 4. Likert scale results from the System Usability Scale survey for the digital system.

© 2021 Nasir-Moin M et al. *JAMA Network Open*.

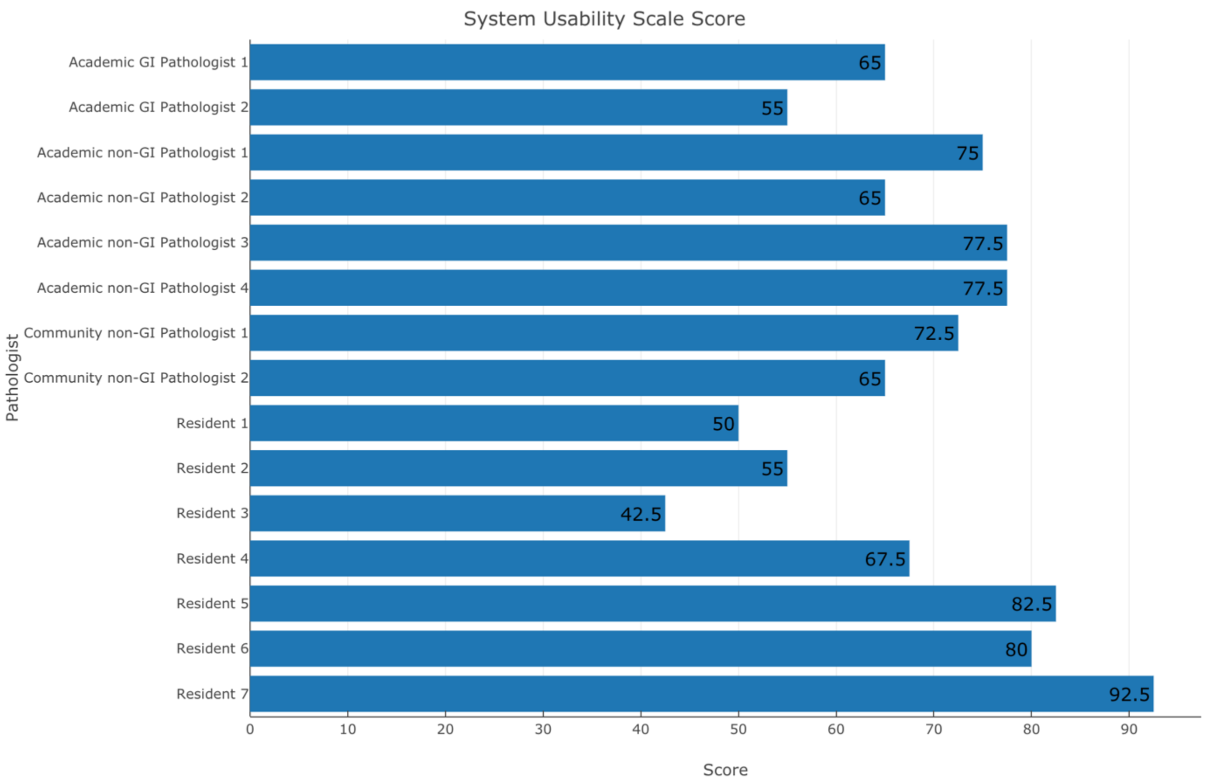

eFigure 5. System Usability Scale Score indicating the overall usability of the digital system for each pathologist

© 2021 Nasir-Moin M et al. *JAMA Network Open*.

Supplemental Tables

Pathologist Type	Institution	Number of Pathologists
Academic GI Pathologist	Dartmouth-Hitchcock Medical Center	2
Academic non-GI Pathologist	Dartmouth-Hitchcock Medical Center	4
Community non-GI Pathologist	Cheshire Medical Center	2
Resident	Dartmouth-Hitchcock Medical Center	7

eTable 1. Crossover study participants. Fifteen pathologists with varying experience levels were enrolled from Dartmouth-Hitchcock Medical Center, an academic institution in Lebanon, NH, and Cheshire Medical Center, a community hospital in Keene, NH.

© 2021 Nasir-Moin M et al. *JAMA Network Open*.

Question
1). "What is your first name?"
2). "What is your last name?"
3). "What is your age?"
4). "What is your gender?"
5). "Which of the following do you identify as?"
6). "Are you board-certified?"
7). "Which specialty are you board-certified in? "
8). "What is your sub-specialty interest?"
9). "How many years have you been in practice? "
10). "Do you practice in an academic or community setting?"

eTable 2. The pre-study survey, which was completed by each pathologist.

© 2021 Nasir-Moin M et al. *JAMA Network Open*.

Statement
1). "I think that I would like to use this system frequently."
2). "I found the system unnecessarily complex."
3). "I thought the system was easy to use."
4). "I think that I would need the support of a technical person to be able to use this system."
5). "I found the various functions in this system were well integrated."
6). "I thought there was too much inconsistency in this system."
7). "I would imagine that most people would learn to use this system very quickly."
8). "I found the system very cumbersome to use."
9). "I felt very confident using the system."
10). "I needed to learn a lot of things before I could get going with this system."

eTable 3. The System Usability Scale is a Likert scale with the following rankings: strongly disagree, disagree, neutral, agree, and strongly agree [2].

© 2021 Nasir-Moin M et al. *JAMA Network Open*.

Statement
1). "In solving or studying the preceding problem I invested…"

eTable 4. The PAAS mental-effort scale is a Likert scale with the following rankings: very, very low mental effort; very low mental effort; rather low mental effort; low mental effort; neither low nor high mental effort; rather high mental effort; high mental effort; very high mental effort; and very, very high mental effort.

© 2021 Nasir-Moin M et al. *JAMA Network Open*.

Statement
1). "Please share any comments (positive or negative), feedback, and suggestions that you might have to improve our tool."
2). "Would you use a version of this tool to sign out cases routinely?"
3). "How has this tool changed your opinions of using artificial intelligence in clinical practice?"

eTable 5. Questions that promoted free-prose feedback.

© 2021 Nasir-Moin M et al. *JAMA Network Open*.

Pathologist	Device	Class	Binary Accuracy	Sensitivity	Specificity
Academic GI Pathologist 1	Microscope	TVA/VA	0.80	0.20	1.00
		SSA	0.93	0.72	1.00
		TA	0.77	0.92	0.72
		HP	0.90	0.96	0.88
	Digital	TVA/VA	0.88	0.52	1.00
		SSA	0.94	0.76	1.00
		TA	0.88	1.00	0.84
		HP	0.94	1.00	0.92
Academic GI Pathologist 2	Microscope	TVA/VA	0.98	0.92	1.00
		SSA	0.97	0.88	1.00
		TA	0.98	1.00	0.97
		HP	0.97	1.00	0.96
	Digital	TVA/VA	0.99	1.00	0.99
		SSA	0.92	0.76	0.97
		TA	0.99	0.96	1.00
		HP	0.92	0.92	0.92
Academic non-GI Pathologist 1	Microscope	TVA/VA	0.79	0.16	1.00
		SSA	0.93	0.76	0.99
		TA	0.79	1.00	0.72
		HP	0.93	0.96	0.92
	Digital	TVA/VA	0.80	0.20	1.00
		SSA	0.91	0.64	1.00
		TA	0.80	1.00	0.73
		HP	0.91	1.00	0.88
Academic non-GI Pathologist 2	Microscope	TVA/VA	0.90	0.60	1.00
		SSA	0.86	0.52	0.97
		TA	0.89	1.00	0.85
		HP	0.85	0.88	0.84
	Digital	TVA/VA	0.97	0.92	0.99
		SSA	0.93	0.80	0.97
		TA	0.94	0.80	0.99
		HP	0.92	1.00	0.89
Academic non-GI Pathologist 3	Microscope	TVA/VA	0.83	0.32	1.00
		SSA	0.87	0.52	0.99
		TA	0.76	0.72	0.77
		HP	0.82	1.00	0.76

© 2021 Nasir-Moin M et al. *JAMA Network Open*.

	Digital	TVA/VA	0.98	1.00	0.97
		SSA	0.89	0.64	0.97
		TA	0.98	0.92	1.00
		HP	0.89	0.92	0.88
Academic non-GI Pathologist 4	Microscope	TVA/VA	0.82	0.28	1.00
		SSA	0.96	0.88	0.99
		TA	0.82	1.00	0.76
		HP	0.96	0.96	0.96
	Digital	TVA/VA	0.82	0.32	0.99
		SSA	0.91	0.68	0.99
		TA	0.83	1.00	0.77
		HP	0.92	0.96	0.91
Community non-GI Pathologist 1	Microscope	TVA/VA	0.90	0.60	1.00
		SSA	0.95	0.80	1.00
		TA	0.90	1.00	0.87
		HP	0.95	1.00	0.93
	Digital	TVA/VA	0.96	0.88	0.99
		SSA	0.93	0.72	1.00
		TA	0.95	0.92	0.96
		HP	0.92	1.00	0.89
Community non-GI Pathologist 2	Microscope	TVA/VA	0.89	0.56	1.00
		SSA	0.91	0.96	0.89
		TA	0.89	1.00	0.85
		HP	0.91	0.68	0.99
	Digital	TVA/VA	0.88	0.52	1.00
		SSA	0.91	0.80	0.95
		TA	0.88	1.00	0.84
		HP	0.91	0.84	0.93
Resident 1	Microscope	TVA/VA	0.95	0.80	1.00
		SSA	0.82	0.28	1.00
		TA	0.94	1.00	0.92
		HP	0.81	0.96	0.76
	Digital	TVA/VA	0.98	0.96	0.99
		SSA	0.86	0.44	1.00
		TA	0.96	0.88	0.99
		HP	0.84	1.00	0.79
Resident 2	Microscope	TVA/VA	0.96	0.88	0.99
		SSA	0.79	0.48	0.89

© 2021 Nasir-Moin M et al. *JAMA Network Open*.

		TA	0.88	0.64	0.96
		HP	0.75	0.76	0.75
	Digital	TVA/VA	0.98	0.92	1.00
		SSA	0.91	0.68	0.99
		TA	0.93	0.80	0.97
		HP	0.86	0.96	0.83
Resident 3	Microscope	TVA/VA	0.80	0.20	1.00
		SSA	0.88	0.64	0.96
		TA	0.76	0.92	0.71
		HP	0.88	0.88	0.88
	Digital	TVA/VA	0.85	0.44	0.99
		SSA	0.81	0.28	0.99
		TA	0.80	0.80	0.80
		HP	0.78	0.96	0.72
Resident 4	Microscope	TVA/VA	0.89	0.56	1.00
		SSA	0.91	0.76	0.96
		TA	0.87	0.92	0.85
		HP	0.91	0.92	0.91
	Digital	TVA/VA	0.89	0.56	1.00
		SSA	0.88	0.56	0.99
		TA	0.88	0.96	0.85
		HP	0.87	0.96	0.84
Resident 5	Microscope	TVA/VA	0.93	0.72	1.00
		SSA	0.90	0.60	1.00
		TA	0.93	1.00	0.91
		HP	0.90	1.00	0.87
	Digital	TVA/VA	0.97	0.88	1.00
		SSA	0.92	0.68	1.00
		TA	0.95	0.92	0.96
		HP	0.90	1.00	0.87
Resident 6	Microscope	TVA/VA	0.81	0.24	1.00
		SSA	0.89	0.72	0.95
		TA	0.68	0.44	0.76
		HP	0.82	1.00	0.76
	Digital	TVA/VA	0.94	0.76	1.00
		SSA	0.91	0.72	0.97
		TA	0.89	0.88	0.89
		HP	0.86	0.84	0.87

© 2021 Nasir-Moin M et al. *JAMA Network Open*.

Resident 7	Microscope	TVA/VA	0.85	0.40	1.00
		SSA	0.75	0.00	1.00
		TA	0.82	0.88	0.80
		HP	0.72	1.00	0.63
	Digital	TVA/VA	0.93	0.76	0.99
		SSA	0.91	0.76	0.96
		TA	0.88	0.84	0.89
		HP	0.90	0.88	0.91

eTable 6. Per class accuracy, sensitivity, and specificity across both devices for each pathologist.

© 2021 Nasir-Moin M et al. *JAMA Network Open*.
